# Association between Hippocampal Volume and Working Memory in 10,000+ 9–10-Year-Old Children: Sex Differences

**DOI:** 10.3390/children8050411

**Published:** 2021-05-18

**Authors:** Shervin Assari, Shanika Boyce, Tanja Jovanovic

**Affiliations:** 1 Department of Family Medicine, Charles R. Drew University of Medicine and Science, Los Angeles, CA 90059, USA; 2 Department of Urban Public Health, Charles R. Drew University of Medicine and Science, Los Angeles, CA 90059, USA; 3 Department of Pediatrics, Charles R. Drew University of Medicine and Science, Los Angeles, CA 90059, USA; shanikaboyce@cdrewu.edu; 4 Department of Psychiatry and Behavioral Neurosciences, Wayne State University, Detroit, MI 48202, USA; tjovanovic@med.wayne.edu

**Keywords:** list sorting working memory, children, right and left hippocampal volume, sex, sex difference

## Abstract

Aim: This study tested sex differences in the association between hippocampal volume and working memory of a national sample of 9–10-year-old children in the US. As the hippocampus is functionally lateralized (especially in task-related activities), we explored the results for the right and the left hippocampus. Methods: This is a cross-sectional study using the Adolescent Brain Cognitive Development (ABCD) Study data. This analysis included baseline ABCD data (*n* = 10,093) of children between ages 9 and 10 years. The predictor variable was right and left hippocampal volume measured by structural magnetic resonance imaging (sMRI). The primary outcome, list sorting working memory, was measured using the NIH toolbox measure. Sex was the moderator. Age, race, ethnicity, household income, parental education, and family structure were the covariates. Results: In the overall sample, larger right (b = 0.0013; *p* < 0.001) and left (b = 0.0013; *p* < 0.001) hippocampal volumes were associated with higher children’s working memory. Sex had statistically significant interactions with the right (b = −0.0018; *p* = 0.001) and left (b = −0.0012; *p* = 0.022) hippocampal volumes on children’s working memory. These interactions indicated stronger positive associations between right and left hippocampal volume and working memory for females compared to males. Conclusion: While right and left hippocampal volumes are determinants of children’s list sorting working memory, these effects seem to be more salient for female than male children. Research is needed on the role of socialization, sex hormones, and brain functional connectivity as potential mechanisms that may explain the observed sex differences in the role of hippocampal volume as a correlate of working memory.

## 1. Introduction

The hippocampus plays a major role in different aspects of learning and memory, including, but not limited to, working memory [1], declarative and procedural memory [2], and short- and long-term memory [3,4]. The size of the hippocampus is linked to higher levels of memory and learning [1,5]. Children and adults with larger hippocampal volumes show higher memory levels [5,6], mainly working memory. Working memory refers to a system for combining the storage and manipulation of information to perform complex cognitive activities [7,8]. Working memory closely reflects the simultaneous involvement of short-term memory and executive functioning in a task. Thus, it involves interacting influences of temporary storage and attentionally based executive control [9].

A high level of working memory is linked with positive outcomes, for example, children’s higher grades and better school performance [10,11,12,13]. Such an effect is replicated frequently and is known to be robust [10,11,12,13].

At the same time, sex differences may exist in cognitive functions such as working memory and executive functioning [14]. Males and females have shown different correlates of working memory and executive control as well [15]. A recent study documented sex-specific activity of brain regions in executive control among participants aged 8–22 years. Using network control theory with diffusion tensor imaging (DTI) data, that study found higher modal controllability in subcortical as well as frontal and parietal cortical regions in females compared to males. In contrast, males showed higher average controllability in subcortical areas and the frontal cortex. This is one of many examples from a large body of papers documenting considerable sex differences in structural brain correlates of executive function [16].

Using Adolescent Brain Cognitive Development (ABCD) data, a national study of brain development with 11,000+ 9–10-year-olds in the United States [17], some studies have established sex differences in executive function and memory [15,18]. Adeli and others [18] showed that a deep learning approach using cerebellar and subcortical brain structures, including the hippocampus, predicted the sex of the ABCD participant with high accuracy while controlling for head size, age, pubertal status, and socioeconomic status (SES). Their deep learning indicator is also closely correlated with working memory. This study suggested that there are major sex differences in brain structures that have implications in working memory [18]. In one ABCD study [15], a high household income better predicted higher list sorting working memory levels in female than male children [15]. In one study, although a higher level of subjective list sorting working memory was associated with better self-rated health for both males and females, self-rated health showed a stronger correlation with objective list sorting working memory in males than females [19]. Another study used the ABCD data and measured executive function using the stop-signal task. Although, in the overall sample, high household income was positively associated with executive function in the children, a stronger effect was found for the effects of high household income on the executive function of female compared to male children [15]. Using the ABCD data, Assari showed sex-specific patterns of sexual maturation and how SES correlates with sex hormones in males and females [20]. These studies suggest that sex may alter the salience of brain structures for working memory in the ABCD Study.

### Aims

In this study, we compared the effects of hippocampal volume on (list sorting) working memory between male and female 9–10-year-old children in the US. While hippocampal volume is expected to be associated with (list sorting) working memory, this effect is expected to differ for females and males. In line with past research [21], males and females show different neurocircuit correlates of various aspects of cognitive function [22], including, but not limited to, working memory and executive function [15,20,23]. Given the years of research on the difference between boys and girls on executive function tasks, we were able to formulate a specific hypothesis regarding the expected direction of the sex difference. Specifically, that means we expected a more salient role of the hippocampus size as a brain structure that predicts the working memory of females compared to males.

Sex differences are reported in the percentage and asymmetry of the principal cranial tissue volumes, which is believed to contribute to sex differences in cognitive functioning [24]. Females have a higher percentage of GM, whereas males have a higher percentage of WM and of CSF. In males, the slope of the relation between cranial volume and GM parallels that for WM, whereas in females, the increase in WM as a function of cranial volume is at a lower rate. In males, the percentage of GM is higher in the left hemisphere, the percentage of WM is symmetric, and the percentage of CSF is higher in the right hemisphere. Females show no asymmetries. However, the regression of cognitive performance and WM volume was significantly steeper in women [24]. Although the left hemisphere is generally dominant in verbal and the right in spatial processing [25], some neuropsychological studies have suggested less hemispheric specialization in women than men [26]. Spatial memory abilities appear to be organized by sex steroids within the first ten days after birth. Interestingly, the effects are accompanied by parallel hormone-induced alterations in hippocampal morphology, in which the granular cell layer of the dentate gyrus (DG) is smaller in adult females and can be masculinized by neonatal treatment with testosterone [27]. Similarly, the volumes of the hippocampal pyramidal cell layers in CA1 and CA3 are greater in males, as are the dendritic fields in CA3 neurons [28]. Moreover, both can be feminized in males by prenatal treatment with antiandrogens and masculinized in females by prenatal administration of testosterone or E_2_ [28]. These data suggest that sex differences in spatial memory may be driven by morphological changes in the hippocampus that are organized by perinatal exposure to E_2_. Hippocampal volumes increase linearly in late childhood/early adolescence in both sexes but then follow different trajectories in males and females during late adolescence such that a continued increase is observed in males, but a slight decrease is found in females [29,30]. Although it is tempting to speculate that sex differences in cognitive function and brain structure result from exposure to pubertal hormones, it is unclear the extent to which these differences relate directly to hormones or rather to some combination of biological and psychosocial alterations [31]. Some tests of hippocampus-dependent spatial learning and memory have shown robust sex differences in adults favoring males [32,33]. Many studies that have applied exogenous hormones [34,35,36] have shown that sex steroid hormones impact hippocampal memory, which differ between females and males [37]. Separate meta-analyses both on human studies and rodent studies have shown sex differences in spatial learning and memory favoring males in young adult humans and rodents [38,39].

However, still, there is a need to expand the current knowledge on sex-specific neural correlates of neurocognitive functions such as working memory [40].

The human hippocampus, although anatomically symmetrical, is functionally lateralized, especially in task-related activities [41]. While the right hippocampus is believed to control spatial information processing, the left hippocampus is believed to be in charge of verbal semantic representations [42,43]. A functional MRI study suggested that the two hemispheres’ hippocampi perform a complementary principle on navigation with places information processing on the right and temporal sequences on the left [44]. In a study of neurosurgical patients, the hippocampus was shown to have lateralized oscillatory patterns in response to memory encoding and navigation [45]. Thus, hippocampal lateralization in spatial cognition is a common phenomenon in higher-order brain function. The hippocampal CA3 contributes to spatial working memory (SWM), but which stage of SWM the CA3 neurons act on and whether the lateralization of CA3 function occurs in SWM are also unknown. Here, we reveal increased neural activity in both sample and choice phases of SWM. Left CA3 (LCA3) neurons show higher sensitivity in the choice phase during the correct vs. error trials compared with right CA3 (RCA3) neurons. LCA3 initiates firing prior to RCA3 in the choice phase. Optogenetic suppression of pyramidal neurons in LCA3 disrupts SWM only in the choice phase. Furthermore, we discover that parvalbumin (PV) neurons, in the MS, projecting to LCA3 impair SWM. The findings suggest that MS^PV^-LCA3 projection plays a crucial role in manipulating the lateralization of LCA3 in the retrieval of SWM [41]. Considering the lateralization of the human hippocampus and its differential engagement in memory, there is a need to differently test associations of the right and left hippocampal volumes with working memory.

## 2. Materials and Methods

### 2.1. Design

This cross-sectional study was a secondary analysis of existing data. We analyzed data from the Adolescent Brain Cognitive Development (ABCD) Study [17,46,47,48,49]. The ABCD is a national children’s brain development study with broad diversity based on race, ethnicity, sex, and SES [46,50].

### 2.2. Original Sample

Participants were mainly recruited from the US school system. The recruitment catchment area of the ABCD dataset included 21 participating sites that encompass more than 20% of the entire population of children in the US. The ABCD Study used local randomization in its sampling and recruitment design [17,46,48,50,51,52,53,54,55,56,57,58,59,60,61,62,63,64,65] to ensure that the sample represents US pre-adolescents. The ABCD recruitment selected schools based on sociodemographic factors such as ethnicity, race, age, sex, SES, and urbanicity to ensure diversity.

### 2.3. Secondary Analysis (Analytical Sample)

This study included 10,093 9–10-year-old children who had data on all study variables, including race, ethnicity, right and left hippocampal volumes, SES, age, sex, and list sorting working memory. Children from all races and ethnic backgrounds were included in the analysis.

### 2.4. Measures

#### 2.4.1. Outcome

Working memory. Our outcome variable, list sorting working memory, measured using the NIH toolbox, was treated as a continuous measure. We used the standardized (for age) rather than the raw score [66]. This variable was available in the NIMH Data Archive (NDA) dataset as well as the Data Exploration and Analysis Portal (DEAP). A higher score was indicative of higher list sorting working memory [66]. Appendix A shows the distribution of the outcome variable in this study.

#### 2.4.2. Predictors

Right and left hippocampal volumes. Predictors were the right and left hippocampal volumes, which were measured using sMRI. The ABCD imaging modalities are well described elsewhere [67]. All participating children in the ABCD Study completed a high-resolution T1-weighted structural MRI scan (1-mm isotropic voxels) with any of the following scanners: Philips Healthcare (Andover, Massachusetts), GE Healthcare (Waukesha, Wisconsin), or Siemens Healthcare (Erlangen, Germany) [17]. All the structural MRI data were processed using FreeSurfer version 5.3.0 [68,69], in line with the standard processing pipelines [17]. The process included the removal of nonbrain tissue, the segmentation of gray and white matter [70], and the parcellation of the cortical and subcortical structures [71]. Every scan session underwent a radiological review. An extended quality control protocol was implemented, which included a visual inspection of T1 images and FreeSurfer outputs for an acceptable quality [72]. Any MRI imaging that did not pass the quality control was excluded. Regions of interest in this study were the right and the left hippocampus. In this analysis, we used the pre-processed volumetric data provided by the ABCD data. Appendix A shows the distributions of the predictor variables in this study. Appendix B shows the models.

#### 2.4.3. Moderator

Sex. Sex, the effect modifier in this study, was a dichotomous variable. Sex was entered as 1 for boys and 0 for girls.

#### 2.4.4. Confounders

Age. Parents reported the age of the children. This variable was continuous in months.

Race. Race, a self-identified variable, was a categorical variable: Black, Asian, Other/Mixed, and White (reference group).

Ethnicity. Ethnicity, self-identified by the parents, was a dichotomous variable: Hispanic vs. non-Hispanic (reference group).

Parental education. Parental education was a 5-level categorical variable. Parents were asked, “What is the highest grade or level of school you have completed or the highest degree you have received?” The categories were less than high school diploma, high school diploma, some college, college graduate, and postgraduate studies.

Parental marital status. The household’s marital status was a dichotomous variable. This variable was coded 1 for married families and 0 for non-married families.

Household income. Household income was an interval measure with a range between 1 and 10, where a score of 1 reflected the lowest and a score of 10 indicated the highest household income level. The item applied to measure household income was the question, “What is your total combined household income for the past 12 months? This should include income (before taxes and deductions) from all sources, wages, rent from properties, social security, disability and veteran’s benefits, unemployment benefits, workman”. Response levels included (1) less than USD 50,000; (2) USD 50,000–100,000; and (3) USD 100,000+.

### 2.5. Data Analysis

**Figure 1:**
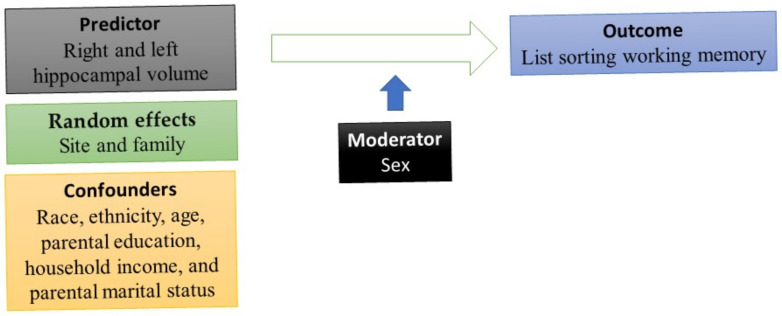
The study schematic diagram (main effects of hippocampus volume, sex, and covariates were tested in *Model 1*; interaction between sex and hippocampus volume was tested in *Model 2*).

DEAP, which is based on the R package, was used for our statistical analyses. To conduct multivariable analysis, two mixed-effect regressions were performed. List sorting working memory was the outcome. Each time, one indicator of right and left hippocampal volume was the predictor (Appendix A). The reason we ran models separately for the right and the left hippocampus was the literature on lateralization of the hippocampus and different implications of the right and the left hippocampus for memory [44], which may differ for males and females [73]. We controlled for race, ethnicity, age, parental education, household income, and parental marital status. Sex was the moderator. All mixed-effect linear regression models were estimated in the pooled sample. We applied mixed-effect regressions because subjects were nested to families that were nested to study sites. Thus, we needed to include family and study sites as random effects (this information is in the Appendix A). *Model 1* was the main effect model. This model was estimated in the absence of the right and left hippocampal volumes by the sex interaction term. *Model 2* was the interaction model. This model added an interaction term between sex and right and left hippocampal volumes. The b coefficient, SE, t values, and *p*-values were reported for each of our parameters in the tested models. We also ruled out multicollinearity between study variables and tested the distribution of our variables and the error terms (residuals). We adjusted for the nature of the data (observations were nested to families to sites), using mixed-effect models. The Appendix A shows the distribution of our outcome, residuals, and quantiles of our outcome variable (list sorting working memory). Figure 1 provides a schematic diagram of the current study and the role of sex as the moderator, age, race, ethnicity, household income, parental education, and family structure as the covariates, working memory as the outcome, and amygdala size as the predictor.

### 2.6. Ethical Aspect

The current study was exempted from a full institutional review board (IRB) review. However, the primary ABCD Study protocol received IRB approval from the University of California, San Diego (UCSD). Participants signed assent, and their parents signed consent [50].

## 3. Results

### 3.1. Descriptives

In Table 1, summary descriptive statistics are shown for both the pooled sample and by each sex. The current study included 10,093 9–10-year-old children, of which 4818 (47.7%) were female, and 5275 (52.3%) were male. Males had higher list sorting working memory than females. Males also showed larger right and left hippocampal volumes than females.

### 3.2. Right and Left Hippocampal Volume and List Sorting Working Memory

Table 2 shows the fit of our models on the effects of right and left hippocampal volumes on list sorting working memory. These models are for the overall sample. For both right and left hippocampal volumes as independent variables, the interaction between sex and right and left hippocampal volumes explained more of the outcome′s variance (from 0.21% to 0.43% change in r square with the inclusion of the interaction between sex and right hippocampal volume, and a change from 0.20% to 0.36% in r square for left hippocampal volume models).

Table 3 depicts the results of two mixed-effect regression models in the pooled/overall sample for right hippocampal volume as the predictor and list sorting working memory as the outcome. *Model 1* explained 0.21% and *Model 2* explained 0.43% of the variation in list sorting working memory. *Model 1* documented a significant and positive association between right hippocampal volume and list sorting working memory overall (b = 0.0013; *p* = 0.001). Other determinants included age, parental education, household income, Black race, and Hispanic ethnicity. *Model 2*, however, showed an interaction between sex and right hippocampal volume on list sorting working memory, suggesting a larger positive association between right hippocampal volume on list sorting working memory for females than males (b = −0.0018; *p* = 0.001).

Table 4 depicts the results of two mixed-effect regression models in the pooled/overall sample with left hippocampal volume as the predictor and list sorting working memory as the outcome. *Model 1* explained 0.20% and *Model 2* explained 0.36% of the variation in list sorting working memory. *Model 1* documented a significant and positive association between left hippocampal volume and list sorting working memory overall (b = 0.0013; *p* = 0.001). Other determinants included age, parental education, household income, Black race, and Hispanic ethnicity. *Model 2*, however, showed an interaction between sex and the left hippocampal volume on list sorting working memory, suggesting a larger positive association between left hippocampal volume on list sorting working memory for females than males (b = −0.0012; *p* = 0.022).

**Figure 2:**
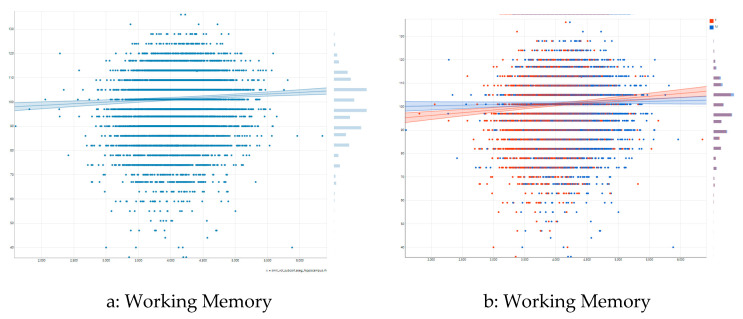
Association between right hippocampal volume and list sorting working memory by sex. (**a**) overall association; (**b**) association by sex.

Figure 2a illustrates the association between right hippocampal volume and list sorting working memory in the pooled/overall sample. As shown by this figure, there is a moderate positive association between right hippocampal volume and list sorting working memory overall. Figure 2b shows differential association by sex. As this figure shows, a steeper positive association exists between right hippocampal volume and list sorting working memory for females than males.

**Figure 3:**
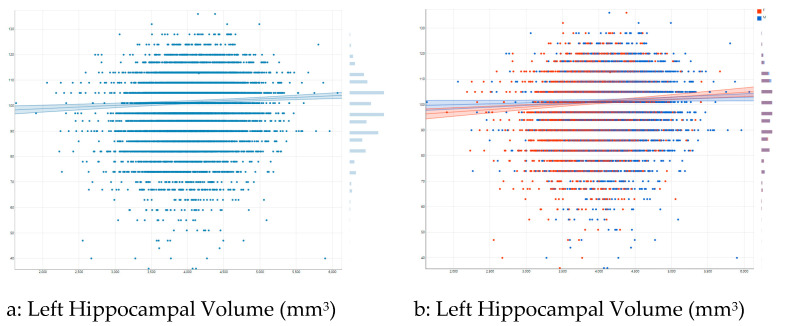
Association between left hippocampal volume and list sorting working memory by sex. (**a**) the overall association; (**b**) association by sex.

Figure 3a shows the association between left hippocampal volume and list sorting working memory in the pooled/overall sample. As this figure shows, there is a moderate positive association between left hippocampal volume and list sorting working memory overall. Figure 3b shows differential association by sex. As this figure shows, a larger positive association exists between left hippocampal volume and list sorting working memory for females than males.

## 4. Discussion

Our study explored sex differences in the association between hippocampal volume and the working memory of children in the US. While a positive association existed between hippocampal volume and children’s working memory, this association was stronger for females than males. The same results were found for right and left hippocampal volumes.

Regarding our main effect, this is consistent with studies that have shown a central role of hippocampal volume in memory [1]. A small hippocampal volume has been shown to be linked to poor memory [1,5]. Individuals with large hippocampal volumes showed higher memory levels than their peers with smaller hippocampal volumes [5,6].

Historically, the hippocampus is known for its contribution to long-term memory. However, there is considerable evidence showing that the hippocampus also contributes to other types of memory such as short-term and working memory. The hippocampus is particularly important for working memory tasks involving relational coding. In a study by Olson and colleagues in 2006 [9], in two experiments, patients with hippocampal lesions had more difficulty remembering the location of a line-drawn object within each of a series of three 3 × 3 matrices, compared to controls. The patients with hippocampal lesions showed a deficit in both studies. Similarly, in the study by Olson and colleagues in 2006 [9], hippocampal patients showed a deficit in delays as short as 4 s in the retention of spatial information, color information, and memory for single faces. Ezzyat and Olson (2008) also showed a deficit in face retention memory over delays of 1 or 8 s in hippocampal patients [74]. Nichols, Kao, Verfaellie, and Gabrieli (2006) found a deficit in face retention memory of a single face after 7 s [75]. Shrager, Levy, Hopkins, and Squire (2008) found a deficit in their H patients after 14 s [76]. In a study by Hannula, Tranel, and Cohen (2006), patients with hippocampal lesions showed impaired recognition memory for both spatial and nonspatial relationships (using faces) even after a brief delay [77]. Our study also shows that the right and left Hs contribute to working memory.

In addition, we found sex differences in the association between hippocampal volume and working memory. Previous research established sex differences in correlates of memory [15]. Neurodevelopment is also sexually dimorphic. The effects of brain structures on neurodevelopment may be specific to sex [78]. Brain structures such as the hippocampus, SES, parenting, brain function, and cognition may show different patterns of association for male and female children. While some brain regions develop faster in males, others may develop faster in females [79,80,81]. A study of 879 youth aged 8–22 years showed sex-specific brain networks that were involved in list sorting working memory. While females had higher modal controllability in frontal, parietal, and subcortical regions, males had higher average controllability in frontal and subcortical regions. Such controllability was associated with lower impulsivity in males but not females. Thus, sex differences exist in the controllability of structural brain networks and their behavioral implications, with such effects possibly being more consequential for males than females [16]. Notably, most prior research included a wide range of ages, which introduces heterogeneity and development-related variability. With respect to understanding sex differences, a wide age range that includes childhood and adolescence also introduces variability in pubertal development [82]. The sex differences in the current study are notable as they were observed in a narrow pre-adolescent age range (9–10 years). Although it is possible that some participants were showing signs of early puberty at this age, the bulk of hormonal changes are typically seen in later adolescence.

Other studied that have used ABCD data have shown a wide range of sex differences that are related to our observation. In one ABCD study [23], sex differences in the association between structural MRI and behavioral inhibition were investigated. While, in the pooled sample, high cortical thickness did not predict behavioral inhibition, sex had a statistically significant interaction with the effect of cortical thickness on children’s behavioral inhibition. A stronger positive effect of high cortical thickness on behavioral inhibition was found for males than females, suggesting that, in the ABCD Study, cortical thickness was a more salient determinant of behavioral inhibition for male than female children [23]. Finally, in another analysis of the ABCD data [15], household income was a better predictor for females′ list sorting working memory than it was for males’ [15]. The study demonstrated that household income is a stronger predictor of list sorting working memory for female than male children. That observation suggested that females living in poverty would show worse list sorting working memory compared to males who live in poverty [15]. These studies show that in the ABCD Study, sex alters how SES, brain structures, and cognitive function correlate. As such, sex is a variable of critical importance in understanding neural development in the ABCD Study.

Environmental inputs, including parenting and SES [83], as well as neurocircuits [23], have shown sex-specific effects on brain structure, brain function, and phenotypic development. A recent study tested whether biological sex shows any statistical interaction with income to explain brain morphology and volume across brain structures in adolescents, cross-sectionally and longitudinally. Overall, income showed effects on cortical gray matter areas, including the cortex and sensorimotor processing areas. These effect sizes were larger for males than for females [84]. In another study, there was a positive association between objective executive memory and self-rated health in males but not females. However, higher levels of subjective executive function were predictive of better self-rated health for both males and females [19]. Mcdermott and colleagues also showed a stronger positive association between SES and cortical surface area for males than females [22]. Whittle and colleagues in 2014 showed that boys’ brain structures may be more sensitive to positive caregiving and parenting [21]. Opposite to these studies, some other research reported stronger correlates of females′ brain function and structure than males. For example, Javanbakht showed SES effects on the hippocampus for females but not males [85], and Kim found that household income was associated with an increased structural brain network efficiency of females but not males aged 6–11 years old [86]. Thus, although sex differences are reported in correlates of brain morphometry, the direction of these sex differences is inconsistent [80].

More research is needed on biological and social mechanisms that may explain why boys and girls differ in the right and left hippocampus volumes’ effect on list sorting working memory. While biological mechanisms should be explored (sex differences), society may also play a role (gender differences). That means parental, behavioral, and psychological causes may interfere with how brain structure impacts boys’ and girls’ opportunities and encounters in society (inside the family and schools). In addition, the intersection of sex, race, place, and class may alter correlates of brain structures for children in the US [87]. This is an emerging field and has some support in the ABCD Study [88,89]. All these complexities require further research.

Our results are important given the role of working memory in academic achievement [90]. Considerable research has connected the higher working memory of children to higher grades and better school performance [10,11,12,13]. The role of working memory as a determinant of school achievement for children has been shown in multiple studies [10,11,12,13,91,92]. The results are also important, given the existing sex differences in various aspects of neurocognitive function including, but not limited to, working memory [93,94] and correlates of such neurocognitive measures [19]. The results reported here may help us better understand how sex differences in working memory emerge and how they contribute to sex differences in academic achievement [95].

While the results are statistically significant, we need to emphasize the difference between statistical and clinical significance. The results may have modest clinical utility because the difference between 96.7 and 97.54 is limited in the context of working memory task performance with a standard deviation of larger than 10. However, at a population level, the results are significant because they are relevant to millions of healthy developing adolescents.

### Limitations

The major limitation of this study is the cross-sectional design. In this study, we only investigated hippocampal volume. A wide range of morphometric features of various cortical and subcortical structures may also have different associations with the working memory of males and females. There is a need for research on sex differences in the impact of functional connectivity of the hippocampus on working memory. We still do not know why hippocampal volume differently influences males’ and females’ working memory. Differential connectivity of the hippocampus with other cortical and subcortical brain regions, sex hormones, or different socialization may explain sex differences in neural correlations of working memory. Future research may also control for more confounders such as total brain volume, physical health, neighborhood SES, or wealth.

## 5. Conclusions

Although we observed a positive association between hippocampal volume and children’s working memory, this link is more pronounced for female than male children. The degree to which a small hippocampal size is associated with poor working memory may differ for males and females. This is important as working memory is a root element of academic and school success among children.

## Figures and Tables

**Table 1:** Descriptive statistics overall and by sex.

Level	All	Female	Male	*p*
*n*	10,093	4818	5275	
	Mean (SD)	Mean (SD)	Mean (SD)	
Age (Month)	118.96 (7.47)	118.79 (7.45)	119.13 (7.48)	0.023
List Sorting Working Memory	97.14 (11.92)	96.70 (11.78)	97.54 (12.04)	<0.001
Right Hippocampus Volume (mm3)	4096.95 (431.43)	3963.32 (396.31)	4219.01 (426.04)	<0.001
Left Hippocampus Volume (mm3)	4048.49 (438.40)	3918.17 (405.83)	4167.51 (433.20)	<0.001
	*n* (%)	*n* (%)	*n* (%)	
Parental Education				
<HS Diploma	363 (3.6)	182 (3.8)	181 (3.4)	0.671
HS Diploma/GED	831 (8.2)	386 (8.0)	445 (8.4)	
Some College	2597 (25.7)	1223 (25.4)	1374 (26.0)	
Bachelor	2673 (26.5)	1273 (26.4)	1400 (26.5)	
Post-Graduate Degree	3629 (36.0)	1754 (36.4)	1875 (35.5)	
Household Income				
<50 K	2889 (28.6)	1398 (29.0)	1491 (28.3)	0.666
≥50 K to <100 K	2896 (28.7)	1382 (28.7)	1514 (28.7)	
≥100 K	4308 (42.7)	2038 (42.3)	2270 (43.0)	
Race				
White	6728 (66.7)	3168 (65.8)	3560 (67.5)	0.279
Black	1449 (14.4)	716 (14.9)	733 (13.9)	
Asian	220 (2.2)	111 (2.3)	109 (2.1)	
Other/Mixed	1696 (16.8)	823 (17.1)	873 (16.5)	
Married Family				
No	3058 (30.3)	1498 (31.1)	1560 (29.6)	0.102
Yes	7035 (69.7)	3320 (68.9)	3715 (70.4)	
Hispanic				
No	8183 (81.1)	3914 (81.2)	4269 (80.9)	0.712
Yes	1910 (18.9)	904 (18.8)	1006 (19.1)	

**Table 2:** The fit of our models on the effects of right and left hippocampal volumes on list sorting working memory.

	Right Hippocampal Volume		Left Hippocampal Volume	
	*Model 1*	*Model 2*	*Model 1*	*Model 2*
*n*	10,093	10,093	10,093	10,093
R-squared	0.12013	0.1211	0.12003	0.12048
ΔR-squared	0.00214	0.00428	0.00203	0.00358
ΔR-squared%	0.21%	0.43%	0.20%	0.36%

**Table 3:** Summary of coefficients on the effects of right hippocampal volume on list sorting working memory.

	*Model 1*				*Model 2*				
	B	SE	*p*	Sig	b	SE	*p*	Sig	
Right Hippocampus Volume	0.0013	0.0003	<0.001	***	0.0023	0.0004	<0.001	***	
Sex (Male)	0.3832	0.2336	0.101		7.7110	2.2045	<0.001	***	
Age (Month)	0.2266	0.0149	<0.001	***	0.2264	0.0149	<0.001	***	
Parental Education (HS Diploma/GED)	2.2258	0.7182	0.002	**	2.2018	0.7178	0.002	**	
Parental Education (Some College)	4.4859	0.6572	<0.001	***	4.4463	0.6570	<0.001	***	
Parental Education (Bachelor)	6.4696	0.6979	<0.001	***	6.4242	0.6976	<0.001	***	
Parental Education (Post-Graduate Degree)	8.1221	0.7066	<0.001	***	8.0632	0.7065	<0.001	***	
Household Income (≥100 K)	2.2578	0.3980	<0.001	***	2.2697	0.3978	<0.001	***	
Household Income (≥50 K to <100 K)	1.4049	0.3540	<0.001	***	1.4072	0.3538	<0.001	***	
Race (Black)	−4.3198	0.3895	<0.001	***	−4.3168	0.3893	<0.001	***	
Race (Asian)	0.3704	0.7799	0.635			0.3697	0.7795	0.635	
Race (Other/Mixed)	−0.5008	0.3239	0.122			−0.5154	0.3238	0.111	
Married Family	0.5099	0.2980	0.0871	#		0.5152	0.2979	0.084	#
Hispanic	−0.8516	0.3428	0.0130	*		−0.8368	0.3428	0.015	*
Right Hippocampal Volume × Sex (Male)						−0.0018	0.0005	0.001	***

# *p* < 0.1, * *p* < 0.05, ** *p* < 0.01, *** *p* < 0.001.

**Table 4:** Summary of coefficients on the effects of left hippocampal volume on list sorting working memory.

	*Model 1*				*Model 2*			
	b	SE	*p*	Sig	b	SE	*p*	Sig
Left Hippocampus Volume	0.0013	0.0003	<0.001	***	0.0019	0.0004	<0.001	***
Sex (Male)	0.4088	0.2325	0.079	#	5.2581	2.1369	0.0139	*
Age (Month)	0.2268	0.0149	<0.001	***	0.2268	0.0149	<0.001	***
Parental Education (HS Diploma/GED)	2.2307	0.7180	0.002	**	2.2162	0.7180	0.002	**
Parental Education (Some College)	4.4717	0.6571	<0.001	***	4.4559	0.6571	<0.001	***
Parental Education (Bachelor)	6.4640	0.6977	<0.001	***	6.4418	0.6977	<0.001	***
Parental Education (Post-Graduate Degree)	8.1204	0.7065	<0.001	***	8.0850	0.7066	<0.001	***
Household income (≥100 K)	2.2580	0.3979	<0.001	***	2.2695	0.3979	<0.001	***
Household income (≥50 K to <100 K)	1.4048	0.3539	<0.001	***	1.4063	0.3539	<0.001	***
Race (Black)	−4.3763	0.3878	<0.001	***	−4.3677	0.3878	<0.001	***
Race (Asian)	0.4359	0.7802	0.576		0.4310	0.7801	0.581	
Race (Other/Mixed)	−0.5013	0.3238	0.122		−0.5097	0.3238	0.115	
Married Family	0.5110	0.2980	0.086	#	0.5140	0.2979	0.084	#
Hispanic	−0.8276	0.3427	0.016	*	−0.8156	0.3428	0.017	*
Left Hippocampal Volume × Sex (Male)					−0.0012	0.0005	0.022	*

# *p* < 0.1, * *p* < 0.05, ** *p* < 0.01, *** *p* < 0.001.

**Figure A1:**
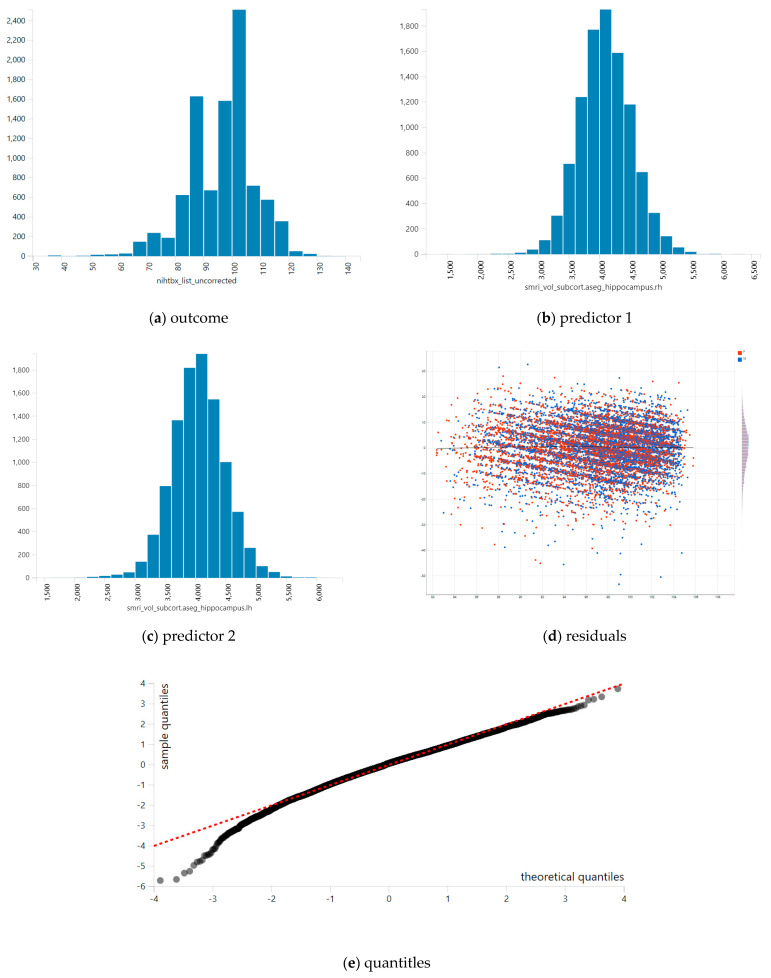
Working memory; (**a**): outcome, (**b**): Right hippocampus, (**c**): Left hippocampus, (**d**): Residuals of the model, (**e**): Quantiles.

## Data Availability

Not applicable.

## Appendix B. Model Formula

	**Right**	**Left**
Model 1	nihtbx_list_uncorrected ~ smri_vol_subcort.aseg_hippocampus.rh + sex + age + high.educ.bl + house-hold.income.bl + race.4level + married.bl + hisp Random: ~(1|abcd_site/rel_family_id)	nihtbx_list_uncorrected ~ smri_vol_subcort.aseg_hippocampus.lh + sex + age + high.educ.bl + house-hold.income.bl + race.4level + married.bl + hisp Random: ~(1|abcd_site/rel_family_id)
Model 2	nihtbx_list_uncorrected ~ smri_vol_subcort.aseg_hippocampus.rh + sex + age + high.educ.bl + house-hold.income.bl + race.4level + married.bl + hisp + smri_vol_subcort.aseg_hippocampus.rh * sex Random: ~(1|abcd_site/rel_family_id)	nihtbx_list_uncorrected ~ smri_vol_subcort.aseg_hippocampus.lh + sex + age + high.educ.bl + house-hold.income.bl + race.4level + married.bl + hisp + smri_vol_subcort.aseg_hippocampus.lh * sex Random: ~(1|abcd_site/rel_family_id)
